# Harm Reduction and Tensions in Trust and Distrust in a Mental Health Service: A Qualitative Approach

**DOI:** 10.1186/s13011-017-0098-1

**Published:** 2017-03-08

**Authors:** Rozilaine Redi Lago, Elizabeth Peter, Cláudia Maria Bógus

**Affiliations:** 1 Present address: Health Science and Sports Center, Federal University of Acre, Room 212, University Campus, Road BR 364, Km 04, Industrial district, 69.920-900 Rio Branco, Acre, Brazil; 2 L. S. Bloomberg Faculty of Nursing, University of Toronto, Toronto, ON Canada; 3 School of Public Health, University of Sao Paulo, São Paulo, Brazil

**Keywords:** Harm reduction, Ethics, Drug users, Trust, Mental health care

## Abstract

**Background:**

People seeking care for substance use (PSCSU) experience deep social and health inequities. Harm reduction can be a moral imperative to approach these persons. The purpose of this study was to explore relationships among users, health care providers, relatives, and society regarding harm reduction in mental health care, using a trust approach rooted in feminist ethics.

**Methods:**

A qualitative study was conducted in a mental health service for PSCSU, and included fifteen participants who were health care providers, users, and their relatives. Individual in-depth and group interviews, participant observation, and a review of patients’ records and service reports were conducted.

**Results:**

Three nested levels of (dis)trust were identified: (dis)trust in the treatment, (dis)trust in the user, and self-(dis)trust of the user, revealing the interconnections among different layers of trust. (Dis)trust at each level can amplify or decrease the potential for a positive therapeutic response in users, their relatives’ support, and how professionals act and build innovations in care. Distrust was more abundant than trust in participants’ reports, revealing the fragility of trust and the focus on abstinence within this setting.

**Conclusion:**

The mismatch between wants and needs of users and the expectations and requirements of a society and mental health care system based on a logic of “fixing” has contributed to distrust and stigma. Therefore, we recommend policies that increase the investment in harm reduction education and practice that target service providers, PSCSU, and society to change the context of distrust identified.

## Background

People seeking care for substance use (PSCSU) experience deep social and health inequities. The perspective of harm reduction can be viewed as an ethical commitment and a moral imperative to approach these persons in such a way that “they are not forced to change and their choices are respected while trust and opportunities to access health care are preserved” ([1, 2], p. 201).

Harm reduction constitutes a fundamental strategy in contemporary mental health care for the benefit of not only PSCSU, but also their relatives and the community. “Harm reduction” has been defined as the combination of policies, programs, pragmatic practices, and practical goals that aim primarily to reduce the adverse health, social, and economic consequences of the legal and illegal use of psychoactive drugs, without necessarily reducing drug consumption [3, 4]. Harm reduction is complementary to prevention and treatment approaches, and empowers drug users to make informed decisions, even with respect to policy-making and program development. Harm reduction emphasizes the humanistic values of dignity, compassion, and nonjudgmental acceptance of people who use drugs. It focuses on users’ access to the highest attainable standard of health care and social services, and is evidence-based and cost-effective, addressing health and social harms associated with illegal drug use, such as soft-tissue infections, blood borne diseases, overdoses, violence, criminalization, and stigma [5–8].

The complexity of mental health care demands that professionals adopt a critical-reflective stance towards users. This perspective should involve acknowledgement of the socio-cultural and political reality of their practice, and be focused more on developing users’ autonomy regarding problem substance use and less on technological interventions, diagnostic labeling, or mere adjustment [2, 9, 10]. The ethical aspects of care in this context imply readiness with the practical, managerial, political, social, and philosophical dimensions of care [2, 9]. Each agent of care must be engaged and open, which requires new dialogue and continuous reflection [9, 10].

A gap exists in the clinical and public health literature regarding ethical dimensions of care for PSCSU. Moreover, there is a lack of understanding regarding the ethical nature of relationships among users and their care providers, family, and the public when harm reduction approaches are employed. Instead, current work “has privileged technical skills and knowledge over discussions about the ability of practitioners to respond to the ethical challenges they encounter in their work” ([11], p. 647). However, each agent of care (researchers, practitioners, policy makers, community members, and PSCSU) has ethical concerns and a role to play in the harm reduction approach.

In the context of ethical dimensions of care for PSCSU, we highlight Baier’s (1985) philosophical work that describes trust as a “reliance on others’ competence and willingness to look after, rather than harm, things one cares about which are entrusted to their care” (p. 259) [12]. Additionally, she highlights the requirement of the goodwill and virtue of the one in whom trust is placed. Baier also describes the importance of thinking critically about “whom,” “why,” and “when” to trust in interpersonal relationships, because trust can lead to tyranny, injustice, and betrayal given the vulnerability of the one who trusts and the corresponding power of the trustee [13]. Trust influences judgments and the therapeutic response in relationships between health professionals and patients [14–16]. Trust also exists in networks, including those with whom we are close and those at a distance, such as policy makers and unknown community members [13]. As such, it is possible to situate the political and normative dimensions of interpersonal relationships within broader interactive structures and contexts and to consider that trust relationships also exist among individuals and groups [16]. In these relationships, personal trust interacts with groups, institutions, and practices based on social structures [15, 17]. Thus, trust often requires more risk for people who are marginalized by mainstream cultures. Additionally, trust in a treatment often involves uncertain efficacy and side effects for the patient, which can be a hard to manage [18]. Trust is a useful concept when examining harm reduction because the nature of relationships formed using this approach are characterized by power differences and the vulnerability of PSCSU that require ethical awareness [2, 11]. Although there have been many useful approaches used to explore the experiences of PSCSU’s, such as the concept of stigma [19–21], the trust offers a novel lens to capture the ethical dimensions of this phenomenon.

Building on the work of Baier (1985, 1986) [12, 13] and other feminist perspectives, McLeod and Sherwin (2000) [17] developed the concept of self-trust, arguing that the internalization of oppression often leads to a lack of self-trust. Without self-trust, persons cannot be fully autonomous. That is, self-trust is needed for persons to trust themselves to make good decisions based on their values, beliefs, and desires, and to act on these decisions and trust their own judgment. Those lacking self-trust often make choices that are not in their best interest and lack competency in exercising their autonomy. McLeod and Sherwin (2000) [17] provide the example of people who use drugs (who often have a history of abuse and oppression) and the challenges they experience trusting themselves and others. They stress the significance of building trust in the relationship between HCP and users, and the need to foster users’ self-trust and autonomy.

In this study, we explored how a trust approach could help to clarify relationships among users, HCP, relatives, and society with respect to harm reduction in a mental health care context for PSCSU of alcohol, crack, and other drugs. Specifically, our research questions were the following: “How are trust and distrust manifested in the relationships that users have with HCP, family, society, and themselves?” and “How are trust and distrust manifested in harm reduction as an approach to drug abuse?”

## Methods

### Design

A critical qualitative research study was conducted [22, 23]. A critical approach demands an active interpretive stance on the part of the researcher in order to recognize power relations, roles, and inter-subjective structures within relationships [22]. Thus, each phase of analysis was grounded in a critical approach, attending to context, social relationships, and the maintenance of an active, interpretive, and reflexive stance [24].

### Participants

Participants were PSCSU, also simply called users (of the service), their relatives, and health professionals directly involved in a specialized public mental health service, located in the North region of Brazil. Health professionals who had worked in an interdisciplinary team for a minimum of six months were invited to participate. The users selected were current clients linked to the service for at least six months, chosen by the health professionals as exemplary cases representing the complexity of needs and demands faced by the team in mental health care. Additionally, we invited one relative of each user chosen to participate in the study. We included persons who were mentioned by practitioners and users as a reference person in the care performed in the service. After obtaining ethical approval from the local university, participants were recruited. They signed an informed consent document, which described the study, risks and benefits, opportunity to withdraw, and measures to maintain confidentiality and data security.

### Description of the setting

The psychosocial care center, or “*Centro de Atenção Psicossocial*” (CAPSad), in Portuguese, constitutes a public health service in Brazil within the mental health care network addressing PSCSU of alcohol, crack and other drugs who are referred either from other community-based health or social services, the criminal justice system, or by self-referral. The service represents a novel landmark intervention in mental health care, providing a low-threshold environment in which the interactions between providers and users are ideally non-judgmental and the care is more accessible. The service aims to minimize harms related to problematic substance use, reducing admissions to psychiatric institutions, and promoting reintegration of these individuals into society by articulation with community service networks [25–28].

Furthermore, the service offers cost-free psychosocial rehabilitation through a multidisciplinary team. This therapy includes individual and group sessions, involving expressivity workshops, crisis intervention, daily or 24/7 shelter, home visits, meetings with users’ family, integration with community services, and follow-up for users in collaboration with other mental health, legal, and social services [26–28].

The majority of users in the service had low socioeconomic status, were unemployed, and were involved in family and legal conflicts. Service reports from 2013 to 2015 presented the majority of users as male (80%), between 25–50 years of age (60%), and having alcohol, cocaine/crack, or polysubstance drug use (90%). The profile of users found in our study follows the description made in other studies involving the same service in different regions of Brazil [26–28]. The average annual number of health care appointments in 2013–2015 was 7,645, of which 9.0% were related to new cases.

### Procedure

Data were generated using multiple sources of qualitative data, including 1) four months of participant observation of the care within the service; 2) individual and group face-to-face interviews with nine HCP (physicians, nurses, psychologists, social workers, physical educators, education technicians, and nurse technicians); 3) individual in-depth face-to-face interviews with three users and three of their relatives, and 4) review of documents such as medical records and service reports. Although a relatively small sample size of participants was used, the breadth and depth of the multiple sources of data collected helped ensure the trustworthiness of the results because of the potential for triangulation [29]. All the interviews conducted with relatives and HCPs were focused primarily on the care experienced by the users, further adding to our understanding of the data obtained from the PSCSU.

Given the abstract nature of the concept of trust, participants were not asked directly about the ethics of trust in the relationship experienced at the service with respect to harm reduction, but instead responded to this open-ended question: “How is the mental health care that is offered and/or that you have experienced in the service?”. This open question made it possible for the interviewer to explore all aspects of care including direct relationships with care providers or PSCSU along with the broader system of mental health and addiction care, allowing issues relating to trust to emerge freely.

Participant observation was conducted in diverse scenarios of care in the service in which HCP and users interacted, such as waiting room, therapeutic groups, case discussion and individual appointments. Thus, the observation focused on how HCP, PSCSU, and other agents interacted in the relationship of care. This stage of the study was carried out by the study’s principal investigator between April and July 2014. Many informal interviews took place, and descriptive and reflexive notes from observation were documented in field notes. Users’ records documented in the service and service reports were photocopied and served as a complementary source of data to interpret the relationship of care described by the participants in the interviews. In addition, the principal investigator, along with one trained research assistant, conducted face-to-face in-depth interviews between August and December 2014. These interviews were audiotaped and took place in a private area within the service, a public space, or at home, depending on the preference and availability of participants.

### Data analysis

After transcription of the audiotapes from interviews, coauthors collaborated throughout the data analysis process to identify and understand emerging themes, key concepts, and their interrelationships involving trust and harm reduction in the relationship of care performed. Field notes, users’ records, and service reports were obtained as an additional source of data. We worked both separately and together comparing common features until we reached consensus regarding the labeling of preliminary codes and categories. Because the focus of this study was on exploring trust and distrust from a feminist perspective, we adopted a critical approach to data analysis. This approach required active interpretation by the researchers to identify power differentials within relationships and the political context of the phenomena [22]. Our shifting of focus back and forth between themes and the research purpose, along with the theoretical underpinnings, meant that we engaged in a “retroductive” process that was both inductive and deductive [24, 29–31]. We created several tentative diagrams of themes and their interrelationships, and ultimately reduced them to three overarching themes representing nested levels of trust and distrust in the treatment, the user, and the self of the user. To ensure rigor, we made certain that the purpose of the research, theoretical assumptions, and method of data analysis were congruent [32]. We also conducted joint meetings with professionals, users, and relatives to discuss the study findings. Thus, participants’ comments during this discussion were incorporated into the reflexive approach taken.

## Results

The theoretical approach allowed us to identify three main themes: 1) (Dis)Trust in the treatment, representing trust and distrust in the harm reduction approach; 2) (Dis)Trust in the user, capturing trust and distrust in PSCSU; and 3) Self-(dis)trust of the user, expressing the perception of trust in the self among PSCSU.

### Trust in the treatment

PSCSU expressed trust in the harm reduction perspective in the treatment, in relation to their relationship with HCP in the process of care. Thus, users indicated the importance of their engagement in and reliance on the care, as well as the opportunity to meet fundamental safety and social needs such as nutrition, hydration, medicine, relationships, recreation, occupation, and instrumental and emotional support, regardless of their drug user status. The extent of their vulnerability was manifested in the fundamental nature of their bodily needs:

“(…) I have been spending time taking “intravenous,” eating, drinking, and then I returned to the street again; they help me a lot.” *User*

“Through the service, I have my normal daily diet and a bath. There were people with whom I could exchange ideas (…).” *User*

In addition, users’ relatives pointed out that the professional team was flexible and open to talking. At the service, they trusted that users would at least have the tools necessary to begin to receive support and treatment in a caring place.

“The team is open to talking, is not inflexible. (…) There [at the service], my father would at least have the potential tools to start treatment.” *Relative*

“(…) He told me that he would go to the service because they would take care of him. He knows that he will find it [care] there; it is a refuge for him.” *Relative*

Moreover, from the perspective of the professional team, the service represents a place of care and support for users where they could rely on the goodwill of the team. According to HCP, the service also helps users to develop strength and demonstrates how to manage everyday problems through a balanced relationship with drugs.

“The user needed help and his relationships were all severed. He had no one to assist him to find help and that's when the service came and made a difference.” *HCP*

“The idea of this care is to strengthen, creating ways to deal with life’s problems.” *HCP*

“We have users who have reached a level of stability in terms of their relationship with the drug; the drug is no longer an obstacle for them to live their lives.” *HCP*

### Distrust of the treatment

In contrast, participants also pointed out aspects of the relationship in the process of care that made them distrust the harm reduction approach to treatment. For users, the idea of treatment normally entailed traditional psychiatric interventions, such as having individual appointments with a physician, taking medicines, being isolated from the community, and experiencing an inpatient regimen of abstinence at a rehab center. Therefore, users felt confused and it took time to understand the type of health care and organizational routine offered through this service.

“I didn’t want to learn about this service. I wanted to move, go to a treatment [based on abstinence in an inpatient rehabilitation center].” *User*

“I was there and I wanted a remedy. I was very nervous, anxious, and angry because they could not give me medication the way I wished.” *User*

Similarly, relatives’ ideas of treatment mainly involved traditional psychiatric interventions and they questioned the team’s professional skills to deal with users. They too occasionally manifested distrust particularly with respect to the team’s capacity to provide the care they believed their relative needed.

“I went there [the service] with him to get his medication. I wanted him to be admitted to another service [inpatient rehabilitation center]. I was so upset.” *Relative*

“I think the team lacks a bit of experience; many of them don’t know how to deal with users.” *Relative*

Furthermore, professional team members expressed frustration related to users’ lapses in abstinence and relapses into intense drug use, demonstrating their lack of complete trust in the ideals of harm reduction, which are generally not abstinence-based:

“The user is trying, but he cannot sustain it, and just falls back.” *HCP*

The care provided to drug users was still focused on specialized mental health services. In this regard, during observation, participants briefly described the need for and challenges associated with integration with primary health care services, which is key to harm reduction approaches. However, public services such as housing, security, citizenship, and social assistance, as well as community organizations that work in the area of mental health were only sometimes integrated into the work of the service, according to observation, interviews, and users’ records. Participants also did not mention in the interviews relevant clinical harm reduction actions, such as counseling and education about safer ways to use drugs; testing for HIV (human immunodeficiency virus), HVC (hepatitis C virus), HVB (hepatitis B virus), and other co-morbidities; the use of contraceptive methods and needle exchange. Moreover, observation and users’ records also showed an insufficient attention to these aspects in the care.

### Trust in the user

Users recognized that abstinence and sobriety were necessary to gain trust, acceptance, and support from their relatives, the community, co-workers, and society, and frequently even from the health professional team and public health services. Indeed, according to users, trust could be gained by making noticed their desire to seek help and to change, thereby revealing the relative power that other people in the lives of PSCSU have and the fragility of trust in these relationships.

“(…) People who are around me need to know that I want to change.” *User*

In addition, as a sign of trust from families, users reported having received support from relatives:

“When I am good, I sleep at my sister’s house. (…) When I go there she always has clothes to give to me; she is always taking care of me.” *User*

### Distrust of the user

References to distrust of users were much more abundant in comparison with those of trust, for all participants’ reports including those of relatives, professionals, and even users themselves.

The distrust content in participants’ reports was related to total abstinence not being a sustainable path for the majority of the users. PSCSU often had alternating cycles of abstinence and relapse during treatment, leading to the difficulties relatives and professionals have in maintaining trust in users.

“I remember my father as a drug user all my life. He spent two weeks on the street and when he wanted, came back. My mother made him soup, washed his clothes, etc. She sent everyone out [us, the children] to let him sleep, for a week straight, to recover and be ready. (…) But he did not want to know [about treatment]; he went back home just to steal something, broke everything, and then left again.” *Relative*

“(…) The addiction is so much bigger than the user.” *HCP*

This unstable situation leads people to see the user as someone who is untrustworthy and is always trying to take advantage of any help. They were not seen as trying to get better, but rather were labeled as “manipulative,” as the HCP pointed out:

“It is typical for users to manipulate thinking: ‘In this moment I am in need and I want to have some help.’ They use this device of needing help to get other things. Many users arrive at the service with this thought: ‘…I’m naked in the street now. I’m broke.’ Then they come to the service, in a gentle and quiet way, to get food, a place to sleep, to call family, trying to restore a connection (…). So, when the family starts to bring clothes and other personal things they need, they think: ‘now I’ve got what I wanted.’” *HCP*

Reinforcing distrust in the user and the professional’s power to define what is normal or good in the PSCSU’s life, a professional described that a user got to the point in the addiction process that he lost his capacity to continue working to provide financial support for the family.

“The user got to the point of his wife having to become the head of the family because everything he received as payment for work was spent on alcohol and other drugs.” *HCP*

In addition, one user expressed negative feelings about the perception of distrust from HCP:

“Most of them see you like a ‘table’ [an object]. They do not place importance on the person, because they think that person has not or will not improve in the future.” *User*

### Self-(Dis)trust of the user

Data regarding self-trust of the PSCSU, while scarce, was expressed by their self-perceptions of hopelessness, stigma, and other harms linked to substance misuse. Furthermore, users realized that their condition had resulted in humiliation and profound suffering for themselves and others:

“(…) Science says that once you are a user [of drugs] you will always be.” *User*

“I already know the suffering that I’ll have and I’ll cause people: humiliation, hunger, cold, loneliness, dirt, everything. I have done this hundreds of times and I cannot stop. I do not believe in myself. (…) My relapses are not ten or twenty; they are five hundred, a thousand. I improve physically, psychologically, and think ‘Now I’m good!’ But I wasn’t, nor I am today and I will never be.” *User*

## Discussion

The nested levels of trust and distrust shown in this study revealed interconnections involving the relationships among HCP, PSCSU, and their relatives regarding a harm reduction approach, trust in users, and trust in the self of the user. Trust/distrust in each of these dimensions can influence access to health care, therapeutic responses in users, engagement of relatives in treatment, and the motivation of professionals to act and build innovations in care [10, 33, 34]. Distrust was more abundant than trust in participants’ reports, revealing the fragility of trust and the focus on abstinence within this setting, despite the harm reduction approach that has been established in mental health policies. These findings are consistent with the ideology of “fixing” the user, a curative approach also called the “getting clean” model of care, through which participants see abstinence as the main goal rather than “the end result of a process, as it is viewed from the Harm Reduction perspective” ([35], p. 6). In this context, stigma is often present and it is hard for all involved to manage such a complex chronic illness that is not easily “fixable” [2, 36, 37]. From an ethical point of view, these tensions underscore differences in the values underlying different approaches to care.

Furthermore, the harm reduction perspective has been developed within the conflicting frameworks of public security (control of drug trafficking and use) and public health (control of demand and reduction of harm for drug users [36, 38]). In addition to this conflict, other social and structural conditions experienced in this scenario increase issues of trust in systems of care, affecting health care utilization, risk accounts, and agency in PSCSU [39–41]. The resulting tension is reflected in the everyday settings of mental health care provision experienced by providers and participants of the service. In this regard, a “reorientation from being an expert on other peoples’ lives towards supporting individuals in their own ways of dealing with problems and struggles” could aid in the development of services targeting users’ needs and preferences, instead of the traditional standard of assessing, adjusting, and fitting users into existing services ([42], p. 3).

Drug users attended the service with a goal to get better, as well as to respond to the expectations of their family and friends. This confirmed that their motivation to seek help involved self-recognition of their poor health and life status, which included experiencing fear and exhaustion, being “disgusted” with themselves, and hitting “rock bottom,” as well as the attitudes of people who mattered to them [43]. Users’ seeking the service, however, did not necessarily want to be abstinent; indeed, they expressed that they were looking for a change and valued the care received with respect to it being welcoming and addressing basic human needs. This finding corresponds to the harm reduction approach, which can accommodate improvement in health without abstinence [11, 44].

The outcomes and success of care were other critical points for participants. Many social opportunities for drug users in treatment are in judgmental and high-threshold settings, in which problem drug use is seen as a choice, and drug users are required to adhere to many strict rules involving complete abstinence and other freedom deprivation aspects [37]. Although we can consider this service as low-threshold [25], stigma, marginalization, and oppression were still present and connected with difficulties in building trust between HCP and drug users in a harm reduction framework [4]. In this regard, through an understanding of therapeutic success as nuanced, low-threshold environments and non-judgmental interactions may allow trust and dialogue to develop among providers and users. Furthermore, as suggested in other related studies, the achievement of de-marginalization, social functioning, mutually trusting relationships between PSCSU and provider, engagement in the program, changes in drug use, and articulation of future goals and plans for users can be used to measure the success of harm reduction-based mental health care [14, 45].

While the harm reduction perspective is criticized as having a neo-liberalism basis, with an over-emphasis on the accountability of the subject and “a prescriptive moralism, based on the duty of citizens to be healthy” [4, p. 5, 46], in the context of public health, it is an effective method to improve access to health care and social justice for users. Because of these benefits, the provision of a harm reduction approach has been viewed as a moral imperative for health care providers [2]. Furthermore, adopting a harm reduction perspective can build mutual trust, which is a path to achieving positive outcomes and change in users’ lives [45].

The ethical dimensions, particularly inequities, inherent in the network of trust are often not acknowledged, including conflicts and invisible oppression related to the international drug market, policies grounded in social control and neoliberalism, and inequities in access to health care [38, 46]. The service described in this research constitutes a novel opportunity for community-based treatment care access in problematic substance use. However, mistrust and discrepancies between care provided and preferences and needs of users continue to challenge access to and stability in treatment [27, 47].

The harm reduction approach employed in this service focused on the provision of housing, access to health services, and meeting basic human needs, which may be helpful to address social determinants of health in this context, in contrast to the much-debated approach related to reducing clinical harm [2]. Additionally, this service should move toward primary health care integration, by offering non-judgmental services under a harm reduction framework, promoting community-based interventions oriented by users’ needs, and engaging communities as partners in psychosocial interventions. Moreover, as other studies suggest, inter-sectoral approaches, integration of community service, continuous training for service providers, and innovations derived from wider health policy reforms can help to address the social determinants of mental health [48, 49].

### Limitations

Several limitations were present in this study. The selection of users was based on HCP’s perceptions of exemplary cases in one specialized mental health care service, which may limit the transferability of the results. In addition, although data were collected by research team members, not linked to the service, and treated confidentially, the process of data collection at the service facility may have affected participants’ responses. Nevertheless, this service represents the main public, cost-free, mental health care strategy available in Brazil, which allowed us to reflect on how PSCSU experience mental health care. Despite these limitations, each insight was grounded in theory and data triangulation, which ensured rigor.

## Conclusions

The study allowed for broad reflection on the harm reduction approach to relationships among PSCSU, their relatives, and HCP; and the challenges they experience. The critical approach used helped us to understand how an ethic of trust is related to care for providers, users, and their relatives in a public mental health care service. Participants’ perceptions of harm reduction approaches within treatment, and the ability of users to sustain themselves in treatment and to develop a better relationship with drugs demonstrated the fragility of trust in this context. The exploration of a harm reduction approach through an ethical lens can strengthen the quality of mental health policies and care provided for PSCSU.

Considering the fragility of trust affecting the relationship of care discussed in our results, as concrete recommendations regarding policy and practice in the area, we suggest: investment in intersectoral engagement approaching policy and practice for people seeking care for substance use, prioritizing harm reduction strategies; the provision of society education towards stigma, marginalization and oppression involving drug use, as well as continuing education for Health Care Providers (HCP) regarding the ethical dimensions of care in this context. In addition, in the therapeutic relationship of care established between providers, users and users’ families, we recommend more dialog, clarity, and structure in expressing expectations and possibilities related to treatment, as well as better visibility and concrete measures of psychosocial improvements reached during treatment for all agents engaged in the care.

## Abbreviations

CAPSad: Psychosocial care center for people seeking care for substance use
HCP: Health care providers
PSCSU: People seeking care for substance use

## References

[1] Cameron A, Abrahams H, Morgan K, Williamson E, Henry L (2016). From pillar to post: homeless women’s experiences of social care. Health Soc Care Community.

[2] Pauly B (2008). Shifting moral values to enhance access to health care: harm reduction as a context for ethical nursing practice. Int J Drug Policy.

[3] Association IHR (2010). What is harm reduction? A position statement from the International Harm Reduction Association.

[4] Keane H (2003). Critiques of harm reduction, morality and the promise of human rights. Int J Drug Policy.

[5] Canadian Nurses Association. Harm reduction and currently illegal drugs: implications for nursing policy, practice, education and research. Discussion Paper. Ottawa: Canadian Nurses Association; 2011.

[6] Stancliff S, Phillips BW, Maghsoudi N, Joseph H (2015). Harm reduction: front line public health. J Addict Dis.

[7] Wilson DP, Donalda B, Shattocka AJ, Wilson D, Fraser-Hurt N (2015). The cost-effectiveness of harm reduction. Int J Drug Policy.

[8] Bruggmann P, Grebely J (2015). Prevention, treatment and care of hepatitis C virus infection among people who inject drugs. Int J Drug Policy.

[9] Carvalho LB, Bosi ML, Freire JC (2008). Dimensão ética do cuidado em saúde mental na rede pública de serviços [Ethical dimension of mental health care within the public health network]. Rev Saude Publica.

[10] Rhodes T, Prodanović A, Žikić B, Kuneski E, Pavićević T, Karadžić D, Bernays S (2008). Trust, disruption and responsibility in accounts of injecting equipment sharing and hepatitis C risk. Health, Risk & Society.

[11] Strike C, Guta A, de Prinse K, Switzer S, Chan CS (2014). Living with addiction: the perspectives of drug using and non-using individuals about sharing space in a hospital setting. Int J Drug Policy.

[12] Baier A (1985). What do woman want in a moral theory?. Noûs.

[13] Baier A (1986). Trust and antitrust. Ethics.

[14] Edland-Gryt M, Skatvedt AH (2013). Thresholds in a low-threshold setting: an empirical study of barriers in a centre for people with drug problems and mental health disorders. Int J Drug Policy.

[15] McLeod C. Trust (2006). Stanford encyclopedia of philosophy.

[16] Peter E, Morgan KP (2000). (2000). Explorations of a trust approach for nursing ethics. Nurs Inq.

[17] McLeod C, Sherwin S, Mackenzie C, Stoljar N (2000). Relational autonomy, self-trust and health care for patients who are oppressed. Relational autonomy: feminist perspectives on autonomy, agency and the social self.

[18] Harris M, Rhodes T, Martin A (2013). Taming systems to create enabling environments for HCV treatment: negotiating trust in the drug and alcohol setting. Soc Sci Med.

[19] Palamar JJ (2012). A pilot study examining perceived rejection and secrecy in relation to illicit drug use and associated stigma. Drug Alcohol Rev.

[20] Palamar JJ (2013). An examination of beliefs and opinions about drug use in relation to personal stigmatization towards drug users. J Psychoactive Drugs.

[21] Tran BX, Vu PB, Nguyen LH, Latkin SK, Nguyen CT, Phan HTT, Latkin CA (2016). Drug addiction stigma in relation to methadone maintenance treatment by different service delivery models in Vietnam. BMC Public Health.

[22] Carspecken P (1996). Critical ethnography in educational research: A theoretical and practical guide.

[23] Creswell JW (2007). Projeto de pesquisa: métodos qualitativo, quantitativo e misto [Reseach Project: qualitative, quantitative and mixed methods].

[24] Peter E, Mohammed S, Simmonds A (2015). Sustaining hope as a moral competency in the context of aggressive care. Nurs Ethics.

[25] Islam M, Topp L, Conigrave KM, Day CA (2013). Defining a service for people who use drugs as ‘low-threshold’: what should be the criteria?. Int J Drug Policy.

[26] Marini M, Schnornberger TM, Brandalise GB, Bergozza M, Heldt E (2013). Quality of life determinants in patients of a psychosocial care center for alcohol and other drug users. Issues Ment Health Nurs.

[27] Gallassi AD, Nakano EY, Wagner GA, Rodrigues MN, Silva MO, Fischer B (2016). Characteristics of clients using a community-based drug treatment service (‘CAPS-AD’) in Brazil: an exploratory study. Int J Drug Policy.

[28] Ribeiro DB, Schneider JF, Terra MG, Soccol KLS, Camillo LA, Plein FAZ (2016). Reasons for attempting suicide among men who use alcohol and other drugs. Rev Gaúcha Enferm.

[29] Mohammed S, Peter E, Gastaldo D, Howell D (2015). Rethinking Case Study Methodology in Poststructural Research. Can J Nurs Res.

[30] Emerson R, Seale C, Gobo G, Gubrium JF, Silverman D (2007). Working with ‘key incidents’. Qualitative research practice.

[31] Kincheloe JL, McLaren P, Denzin NK, Lincoln Y (2005). Rethinking critical theory and qualitative research. Qualitative research.

[32] Melia KM, Bourgeault I, DeVries R, Dingwall R (2010). Recognizing quality in qualitative research. Handbook of qualitative research.

[33] Whetten K, Leserman J, Whetten R, Ostermann J, Thielman N, Swartz M, Stangl D (2006). Exploring Lack of Trust in Care Providers and the Government as a Barrier to Health Service Use. Ame J Public Health.

[34] Jauffret-Roustide M, Cohen J, Poisot-Martin I, Spire B, Gossop M, Carrieri MP, the MANIF 2000 Study Group (2012). Distributive sharing among HIV–HCV co-infected injecting drug users: the preventive role of trust in one’s physician. AIDS Care.

[35] Peterson J, Mitchell SG, Hong Y, Agar M, Latkin C (2006). Getting clean and harm reduction: adversarial or complementary issues for injection drug users. Cad Saúde Pública.

[36] Garcia MLT, Leal FX, Abreu CC (2008). A política antidrogas brasileira: velhos dilemas [Brazil’s anti-drugs policies: old dilemmas]. Psicologia & Sociedade, Porto Alegre.

[37] Ronzani TM, Higgins-Biddle J, Furtado EF (2009). Stigmatization of alcohol and other drug users by primary care providers in Southeast Brazil. Soc Sci Med.

[38] Bastos FI (2012). Structural violence in the context of drug policy and initiatives aiming to reduce drug-related harm in contemporary Brazil: A review. Subst Use Misuse.

[39] Ostertag, S., Wright, B., Broadhead, R. S., Altice, F. Trust and other characteristics associated with health care utilization by injection drug users. J of Drug Issues. 2006;36:953–974.

[40] Treloar C, Rance J, on behalf of the ETHOS Study Group (2014). How to build trustworthy hepatitis C services in an opioid treatment clinic? A qualitative study of clients and health workers in a co-located setting. Int J Drug Policy.

[41] Treloar C, Rance J, Yates K, Mao L (2016). Trust and people who inject drugs: The perspectives of clients and staff of Needle Syringe Programs. Int J Drug Policy.

[42] Borg M, Kristiansen K (2004). Recovery-oriented professionals: Helping relationships in mental health services. J Ment Health.

[43] Andrews D, Kramera R, Klumper L, Barrington C (2012). A qualitative exploration of individuals’ motivators for seeking substance user treatment. Subst Use Misuse.

[44] Kothari G, Hardy G, Rowse G (2010). The therapeutic relationship between therapists and substance-using clients: a qualitative exploration. J Subst Use.

[45] Lee HS, Zerai A (2010). “Everyone deserves services no matter what”: defining success in harm-reduction-based substance user treatment. Subst Use Misuse.

[46] Moore D, Fraser S (2006). Putting at risk what we know: reflecting on the drug-using subject in harm reduction and its political implications. Soc Sci Med.

[47] Morgan K, Lee J, Sebar B (2015). Community health workers: a bridge to healthcare for people who inject drugs. Int J Drug Policy.

[48] Islam M, Topp L, Day CA, Dawson A, Conigrave KM (2012). The accessibility, acceptability, health impact and cost implications of primary healthcare outlets that target injecting drug users: a narrative synthesis of literature. Int J Drug Policy.

[49] Ventevogel P (2014). Integration of mental health into primary healthcare in low-income countries: avoiding medicalization. Int Rev Psychiatr.

